# Multiview video plus depth transmission via virtual-view-assisted complementary down/upsampling

**DOI:** 10.1186/s13640-016-0119-4

**Published:** 2016-04-29

**Authors:** Zhi Jin, Tammam Tillo, Jimin Xiao, Yao Zhao

**Affiliations:** Department of Electrical and Electronic Engineering, Xi’an Jiaotong-Liverpool University, Ren Ai Road 111, Suzhou, 215123 People’s Republic of China; Department of Electrical Engineering and Electronics, University of Liverpool, Merseyside L69 3BX, Liverpool, UK; Institute of Information Science, Beijing Jiaotong University, No.3 Shangyuancun Haidian District, Beijing, People’s Republic of China

**Keywords:** Low bit rate video transmission, Video coding, Multiview video plus depth (MVD), Depth map, DIBR, Virtual view

## Abstract

Multiview video plus depth is a popular 3D video format which can provide viewers a vivid 3D feeling. However, its requirements in terms of computational complexity and transmission bandwidth are more than that of conventional 2D video. To mitigate these limitations, some works have proposed to reduce the amount of transmitted data by adopting different resolutions for different views, and consequently, the transmitted video is called mixed resolution video. In order to further reduce the transmitted data and maintain good quality at the decoder side; in this paper, we propose a down/upsampling algorithm for 3D multiview video which systematically takes into account the video encoder and decoder. At the encoder side, the rows of the two adjacent views are downsampled following an interlacing and complementary fashion, whereas, at the decoder side, the discarded pixels are recovered by fusing the virtual view pixels with the directional interpolated pixels from the complementary downsampled views. Moreover, the patterns of the texture surrounding the discarded pixels are used to aid the data fusion, so as to enhance edges recovery. Meanwhile, with the assistance of virtual views, at the decoder side, the proposed approach can effectively recover the discarded high-frequency details. The experimental results demonstrate the superior performance of the proposed framework.

## Introduction

The development of 3D technologies and communication networks makes 3D video applications increasingly popular. An example of this technology is 3D multiview television which allows to cover a wide view angle of the scene. However, delivering a large number of high-quality views to end users is a challenging task due to the limitations of data transmission and storage capacity [1]. Therefore, some advanced video coding standards have been proposed to compress video data, such as H.264/MVC [2] and HEVC [3–6]. Besides that, some data representation formats for the 3D multiview data have been adopted for efficient representation. One widely accepted format is the multiview video plus depth (MVD) format [7], which consists of textures and the associated per-pixel depth data (the latter describes the geometric relationship between objects in the scene and the capturing cameras [8]). Since this format allows any intermediate view within a certain range to be generated, with the assistance of the depth-image-based rendering (DIBR) technique [9]. Therefore, it can, to a large extent, reduce the number of transmitted views. However, the required data of 3D multiview video is still very large.

Because of the above reasons, many works have focused on reducing the amount of transmitted data at the encoder side and recovering it at the decoder side for low bit rate transmission [10–12]. Additionally, given that depth maps consist of large homogeneous areas, they require less transmission bit rate compared with texture [13]. Therefore, the reduction and recovery of texture data have drawn more attention than that of the depth data. In order to speed up the encoding process and reduce the overall bit rate, in [14], Garcia et. al adopted a low-resolution (LR) and full-resolution (FR) frames mixed video sequence. Based on this, a mixed resolution coding approach was proposed where the first *M* frames in the sequence were encoded at FR and the rest frames were coded at LR. Mixed resolution (MR) view frameworks have been proposed in [15, 16] for multiview video coding, where at least one view is coded at LR, while the others are coded at FR. These frameworks can reduce the amount of transmitted and stored data in comparison with the full FR framework. In [17, 18], a MR-MVD framework was adopted at the encoder side and 3D warping-generated virtual views were utilized to recover FR frames from the LR frames at the decoder side. With proper reconstruction algorithms at the decoder side, MR-MVD framework can well enhance the overall transmission efficiency.

In terms of reducing the transmission and storage data size, for stereoscopic video, the MR format can save 3/8 transmitted texture data compared with FR format, while downsampling both views by half can reduce the amount of transmitted texture data to 1/2 of the original format. Furthermore, the neighboring views have the same frame size which is suitable for the MVC coding approach. Two frame-compatible coding frameworks for stereoscopic video were proposed in [19, 20]. In [19], two views were decimated to half of their original size by downsampling filters, which were designed based on frame contents and the targeted interpolation coefficients. At the decoder side, the reconstructed frames were demultiplexed and interpolated into the full resolution. Since the frame downsampling pattern at the encoder side was evaluated as a function of the interpolation method, it can, to a large extent, reduce the interpolation errors. Unfortunately, this paradigm is not suitable for video applications because the downsampling pattern is frame dependent which means that the temporal redundancy cannot be efficiently removed by the video encoder. Thus, for video applications, there is a need to use temporally static downsampling patterns. In [20], an opposite parity packing arrangement for stereoscopic video was proposed to multiplex the two views. At the decoder side, the optimal disparity vector for each block was obtained from calculating the smallest differences between the matched blocks in coarsely interpolated FR left view and right view. However, in this work, some theoretical supports for the proposed view packing arrangement were missing. Moreover, although both [19, 20] had shown superior performance than other state-of-the-art frame-compatible coding frameworks, they mainly focus on stereoscopic video which may not be optimal for multiview or MVD video.

**Fig. 1:**
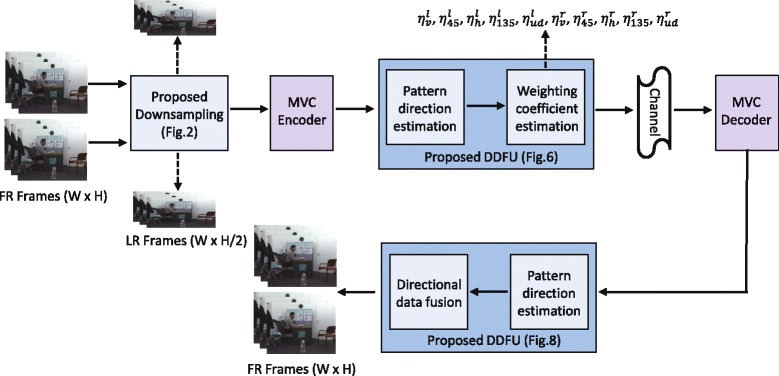
Framework of the proposed down/upsampling method. Framework of the proposed down/upsampling method for a stereo video

In this paper, a *systematical* down/upsampling framework for MVD video is proposed to enhance the coding performance at low bit rate (Fig. 1). In the proposed downsampling approach, the rows of two adjacent texture views are downsampled following an interlacing and complementary pattern, before compression. The aim of this downsampling approach is to facilitate the upsampling at the decoder side while exploiting the feature of 1D DIBR. The LR views will be upsampled by fusing the virtual view pixels with directional interpolated pixels with the aid of the pattern of the texture surrounding the discarded pixels. This approach has two benefits. Firstly, the high frequency information contained in one LR view can be properly utilized to upsample the other LR view through the generated virtual views. Secondly, the proposed directional interpolation approach can overcome the limitation of the virtual-view-based upsampling mechanism, which suffers in the areas corresponding to depth map discontinuity. Hence, by taking advantage of these two strategies, the discarded pixels can be recovered effectively. Experimental results have shown that the proposed algorithm achieves superior performance with respect to the filter-based interpolation algorithms and other state-of-the-art algorithms. The proposed upsampling approach will be named directional data fusion upsampling (DDFU) throughout this paper.

The rest of this paper is organized as follows. Section 2 describes the details of the proposed downsampling algorithm, and the upsampling algorithm is introduced in Section 3. The generalization of the proposed method is presented in Section 4, and experimental results are presented in Section 5. Finally, the conclusions are in Section 6.

## Proposed interlacing-and-complementary-row-downsampling

**Fig. 2:**
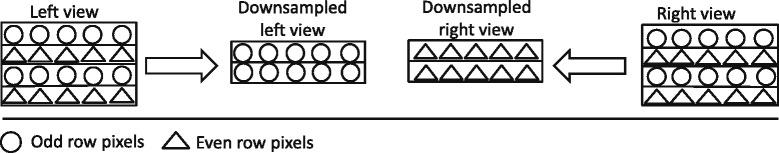
The proposed downsampling method. The proposed interlacing-and-complementary-row-downsampling process for a stereo video

A proper downsampling approach for multiview video needs to take into account the fact that different views cover almost the same scene. Hence, between neighboring views, there is considerable amount of inter-view redundancy. In this work, an interlacing-and-complementary-row-downsampling method is proposed by taking the features of proposed upsampling and multiview video into account, as shown in Fig. 2.

**Fig. 3:**
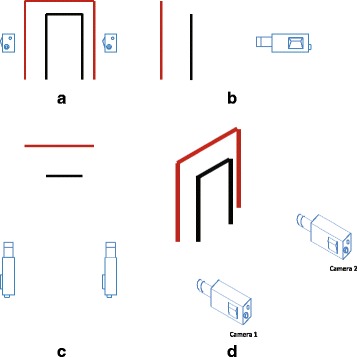
Captured scene description. **a**, **b**, and **c** show the front, side, and top view of the stereoscopic orthographic projection of uneven bar structure viewed by two cameras in a parallel configuration setting as depicted in (**d**)

**Fig. 4:**
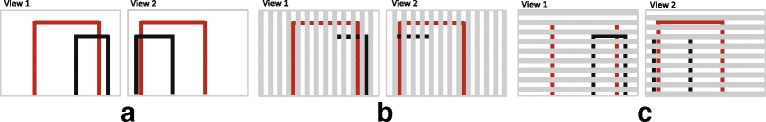
The outputs of different downsampling methods. **a** the left side and right side of each frame shown the captured scene by the corresponding cameras without downsampling, respectively; **b** the output of the vertical downsampling method (i.e., column-wise downsampling); **c** the output of the interlacing and complementary row-wise downsampling method

**Fig. 5:**
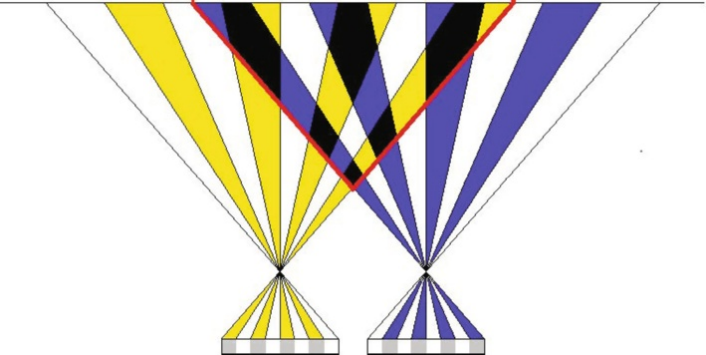
The *top view*of the prospective projection. The *top view* of the prospective projection of a scene using a pinhole camera model for the column-wise downsampling approach; area which could be viewed by both cameras (before the downsampling process) is enclosed in *red*; *yellow* bands indicate areas that cannot be seen in view 1 due to the column-wise downsampling approach; unviewed areas in view 2 due to the downsampling are indicated by *blue*. Areas depicted in black cannot be viewed in both views, those are called “blind areas”

In the view multiplexing approaches, the generated sequence is from the mapping of two downsampled views. The process of multiplexing is carried out before the video encoding stage. There are variety of options for both the downsampling and view combination [21]. Due to inter-view redundancy, interlacing-and-complementary downsampling approaches could maintain more information than the non interlacing and complementary ones. In the following parts and aided with a graphical example, three downsampling approaches will be compared. In these three scenarios, we assume that two calibrated cameras in a parallel configuration setting and the same image plane (the most common camera configuration) are used to record an uneven bar structure (similar to the artistic gymnastics apparatus), as shown in Fig. 3d. Figure 3a–c shows the front, side, and top view of the stereoscopic orthographic projection of the scene, respectively. The viewed scene of the first and second cameras is shown in Fig. 4a. The output of the vertical interlacing and complementary downsampling approach (i.e., column-wise downsampling) is shown in Fig. 4b, where the gray areas indicate the “discarded areas” during downsampling process. It is possible to see that the left black bar of the uneven bar structure is missing in both views. Hence, neither intra-view or inter-view interpolation can help to recover this part. This is because the column-wise downsampling approach causes some “blind areas,” where objects cannot be seen in any of the two views. The “blind areas” could be easily seen in Fig. 5, which shows the top view of the prospective projection of a scene with two pinhole cameras. The area enclosed by red lines can be viewed by both cameras, whereas, the yellow and blue bands indicate discarded areas in view 1 and view 2, respectively, due to the column-wise downsampling. Some areas (indicated by black) inevitably end up being discarded in both views; thus, any object falling in any of these black areas cannot be recovered by inter-view interpolation, and consequently, these areas are called “blind areas”.

Compared with column-wise downsampling, the output of row-wise one is shown in Fig. 4c. It indicates that the proposed interlacing-and-complementary-row-downsampling will almost always guarantee that the object can be seen in the rows of one of the two views, except for some small objects with a one-pixel-width projection size in the camera plane. Nevertheless, the probability of this situation is low, and it also happens for the column-wise downsampling approach. Consequently, this row-wise downsampling can better exploit the warping feature of the DIBR technique and, as a result, can enhance the upsampling performance.

The chessboard downsampling approach can be regarded as the combination of row- and column-wise ones. It is able to achieve highest intra-view upsampling performance, since each to-be-filled pixel has four adjacent pixels in both horizontal and vertical directions which provide more information during interpolation. However, the chessboard pattern usually requires a comparatively higher bit rate due to low spatial and temporal correlations [19]. Furthermore, for each row of the chessboard downsampled views, it is possible to notice that the top view of the prospective projection of a scene is similar to the one shown in Fig. 5. Therefore, it could be conjectured that the chessboard approach also suffers from some “blind areas”; thus, its performance is better than the column-wise approach while being worse than the row-wise approach.

## Virtual view-assisted directional data fusion upsampling

In order to reduce the required resources and the amount of transmitted data, downsampling of the texture sequences is performed before the compression stage. In this paper, motivated by the findings in Section 2, the downsampled texture frames are generated by discarding the even rows in the left view and the odd rows in the right view of the stereo video, respectively.

**Fig. 6:**
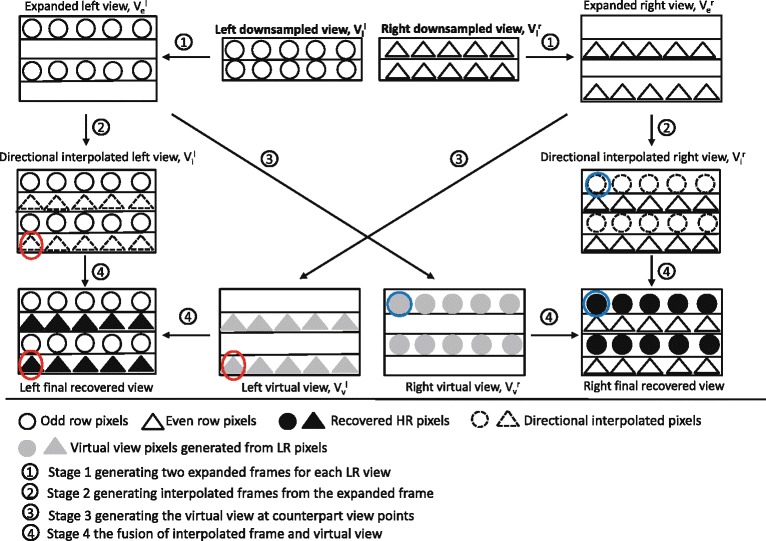
The proposed discarded pixels recovery process

Let the left and right FR frames be defined as $\mathbf {V}_{f}^{l}$ and $\mathbf {V}_{f}^{r}$, respectively, with size *W*×*H*, and the downsampled left and right LR frames as $\mathbf {V}_{l}^{l}$ and $\mathbf {V}_{l}^{r}$, respectively, with size *W*×*H*/2. Figure 6 shows the main stages of the proposed FR recovery mechanism. The downsampled views are expanded to their original size with the positions of the discarded pixels left empty (this stage is indicated by ① in Fig. 6). The expanded left view is represented by $\mathbf {V}_{e}^{l}$ where ${V_{e}^{l}}(2n,m)=0, 1\le n \le H/2, 1\le m\le W$, whereas, the expanded right view is represented by $\mathbf {V}_{e}^{r}$ where ${V_{e}^{r}}(2n-1,m)=0, 1\le n \le H/2, 1\le m\le W$. Then, in the second stage indicated by ② based on the direction estimation results, a directional interpolation method is used to generate the corresponding interpolated frames, and these are denoted by $\mathbf {V}_{i}^{l}$ and $\mathbf {V}_{i}^{r}$ for the left and right views, respectively. Meanwhile, in the third stage ③, the DIBR technique is applied on the expanded frames using the corresponding depth maps in order to generate the virtual views at the counterpart viewpoints, i.e., the left side virtual view $\mathbf {V}_{v}^{l}$ is generated by the right side expanded view. As a consequence, all the even rows in the left virtual view are warped from the even rows in the right view. Similarly, for the right virtual view, all the odd rows are warped from the odd rows in the left view. Therefore, based on the above design which aims to make the recovery of discarded pixels work in synergy with the downsampling stage, the virtual view becomes a potential source of information to efficiently recover the discarded pixels. Virtual views and directional interpolated views as the outputs of the two parallel stages,② and ③, are fused to generate the final FR frames at stage ④. This fusion process is driven by the pattern direction of the texture around each of the discarded pixels, so as to exploit the potential of stages ② and ③.

### PCA-based pattern direction estimation

Knowing the dominant direction of the texture surrounding each discarded pixel allows better exploitation of the virtual and interpolated frames in recovering the discarded pixels. For example, texture patterns with horizontal edges usually cannot be accurately estimated from their upper and lower neighbors. Hence, exploiting the virtual view pixels can greatly help to recover such pixels.

To get the pattern direction, in this work, a principal components analysis (PCA)[22]-based method is used. This approach evaluates the gradients of the surrounding pixels for each discarded pixel, and then, the dominant direction is determined [23], where PCA can be implemented by evaluating the singular value decomposition (SVD) [24] of the data.

In general, the gradient at *V*(*x,y*) can be obtained by ∇*V*(*x,y*)=[*∂V*(*x,y*)/*∂x*,*∂V*(*x,y*)/*∂y*]^*T*^, and this could be approximated for discrete applications as

(1) $$ \nabla{V(x,y)} \approx \left (\begin{array}{c} \frac{1}{2}(V(x+\Delta,y)-V(x-\Delta,y)) \\ \frac{1}{2}(V(x,y+\Delta)-V(x,y-\Delta)) \end{array} \right)  $$

*Δ*=1 offers the best approximation, however, taking into account that half of the rows are discarded, then *Δ* needs to be 2 while evaluating the gradients of the surrounding pixels of a discarded pixel. This ensures that *V*(*x*+*Δ*,*y*), *V*(*x*−*Δ*,*y*), *V*(*x,y*+*Δ*), and *V*(*x,y*−*Δ*) are available^1^.

**Fig. 7:**
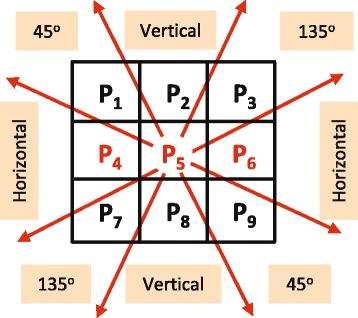
Missing pixels classification based on texture pattern directions. The overlapping window centered at the discarded pixel *p*_5_. The dominant pattern direction will be categorized into five groups. In this figure, only the remarkably dominant patterns are shown which are horizontal, 45° diagonal, vertical, and 135° diagonal directions

It is worth noticing that the horizontal neighbors (i.e., left and right neighbors) of the discarded pixels are unavailable; therefore, the dominant direction for each discarded pixel will be inferred from the four corner pixels of a 3×3 overlapping window centered at the discarded pixel. For example, the discarded pixel *p*_5_, in Fig. 7, has two discarded neighbors, namely *p*_4_ and *p*_6_, so in order to maintain an equivalent number of neighbors and symmetric structure around *p*_5_, the two pixels *p*_2_ and *p*_8_ will not be taken into account while evaluating the dominant pattern direction. In other words, only the gradients of the corner pixels *p*_1_,*p*_3_,*p*_7_, and *p*_9_ will be evaluated^2^. The gradients of the surrounding pixels of the discarded pixel at position (*x,y*) will be then arranged into a 4×2 matrix **G**, as follows:

(2) $$ \mathbf{G}= \left[ \begin{array}{c} \nabla{V(x-1,y-1)}^{T} \\ \nabla{V(x-1,y+1)}^{T} \\ \nabla{V(x+1,y-1)}^{T}\\ \nabla{V(x+1,y+1)}^{T} \end{array} \right]  $$

The SVD of the matrix **G** will be computed as **G**=**USV**^**T**^, where **S** is a 4×2 diagonal matrix and the ratio of the diagonal elements in **S** (i.e., *S*_11_/*S*_22_) represents the energy of the dominant gradient. **U** and **V** are orthogonal matrices with size 4×4 and 2×2, respectively, and the angle of dominant gradient is *θ*= arctan(*ν*_21_/*ν*_11_). For the remarkably dominant gradient (i.e., *S*_11_/*S*_22_≥*Th* where *Th* is a threshold to define the remarkably dominant gradient), this angle will be used to determine the pattern directions of the discarded pixel, which are horizontal, 45° diagonal, vertical, and 135° diagonal directions as shown in Fig. 7. For the pixels from texture uniform areas whose energy in all four directions is almost equal, there is no remarkably dominant directional pattern (i.e., *S*_11_/*S*_22_<*Th*), will be classified into the “undefined” direction category. This process will be carried forward at both the encoder and decoder sides.

### Weighting coefficient estimation of directional data fusion

Since all of the discarded pixels are classified into five categories: *horizontal*, 45° *diagonal, vertical*, 135° *diagonal*, and *undefined direction*, the directional interpolated frames $\mathbf {V}_{i}^{l}$ and $\mathbf {V}_{i}^{r}$ are generated based on this classification. The *horizontal pixels* are the average of corresponding four nearest corner pixels, and the *undefined directional pixels* are recovered by vertical interpolation, since the vertical neighbors are the closest to the discarded pixels. For the other three directions’ pixels, they are recovered by linearly interpolation along the pattern direction. In the fourth stage of the proposed upsampling algorithm, the discarded pixels are recovered by fusing the interpolated pixels with the virtual view pixels in order to exploit the advantages of both types of approach and to compensate the compression distortion.

To recover the discarded information, at the fusion stage, each discarded pixel is filled by a weighted average of the counterpart pixels in *V*_*v*_ and *V*_*i*_ as shown:

(3) $$ \hat {V}^{l}(2n,m) = \eta^{l} {V_{i}^{l}}(2n,m) + (1- \eta^{l}) {V_{v}^{l}}(2n,m)  $$

The value of the weighting coefficient, *η*^*l*^, is in the range [0,1]. This value, in theory, should be evaluated for each missing pixel, and it determines the relative contribution of the directional interpolated pixel with respect to the virtual view pixel. The fusing coefficients could be obtained by minimizing the L2 distance between the recovered pixels and their counterpart original pixels, as follows.

(4) $$ \sum_{m=1}^{W}\sum_{n=1}^{H/2}\left(\hat {V}^{l}(2n,m) - {V_{f}^{l}}(2n,m)\right)^{2}  $$

Holes and disoccluded areas in the virtual views are excluded during the fusion process and in these areas; the discarded pixels are directly recovered by directional interpolation. Since the original FR frame is only available at the encoder side, this means that all the fusing coefficients need to be transmitted for each frame to the decoder side. Obviously, this makes the pixel-by-pixel estimation of the weighting coefficient impractical. Hence, in this paper, a direction-mask-based weighting coefficient estimation is proposed, which can hugely reduce the transmitted side information. At the encoder and decoder sides, all the discarded pixels will be classified into five categories and respectively represented by five binary masks *M*_*h*_, *M*_45_, *M*_*v*_, *M*_135_, and *M*_*ud*_. In this way, the binary value “1” in *M*_*h*_ indicates that the discarded pixel in that position has a horizontal texture pattern; in this case, the same position in *M*_*v*_, *M*_45_, *M*_135_, and *M*_*ud*_ will have “0” binary value. For each direction, one weighting coefficient will be estimated by (4). Therefore, Eq. (3) could be rewritten in matrix format, while taking into account the five pattern categories, as follows:

(5) $$ \begin{array}{ll} \hat{\mathbf{V}}^{l} &= {\eta_{h}^{l}} \mathbf{{M_{h}^{l}}}.* \mathbf{V}_{i}^{l} + (1- {\eta_{h}^{l}}) \mathbf{{M_{h}^{l}}}.* \mathbf{V}_{v}^{l} \\ &\quad+\eta_{45}^{l} \mathbf{M_{45}^{l}}.* \mathbf{V}_{i}^{l} + (1- \eta_{45}^{l}) \mathbf{M_{45}^{l}}.* \mathbf{V}_{v}^{l} \\ &\quad+{\eta_{v}^{l}} \mathbf{{M_{v}^{l}}}.* \mathbf{V}_{i}^{l} + (1- {\eta_{v}^{l}}) \mathbf{{M_{v}^{l}}}.* \mathbf{V}_{v}^{l} \\ &\quad+\eta_{135}^{l} \mathbf{M_{135}^{l}}.* \mathbf{V}_{i}^{l} + (1- \eta_{135}^{l}) \mathbf{M_{135}^{l}}.* \mathbf{V}_{v}^{l}\\ &\quad+ \eta_{ud}^{l} \mathbf{M_{ud}^{l}}.* \mathbf{V}_{i}^{l} + (1- \eta_{ud}^{l}) \mathbf{M_{ud}^{l}}.* \mathbf{V}_{v}^{l} \end{array}  $$

where $\hat {\mathbf {V}}^{l}$ denotes the recovered frame. The operation.∗ represents the element-by-element multiplication of two matrixes. The optimal weighting coefficient for each direction can be obtained applying (4) on each direction.

Given that the encoder and decoder work on the same set of data to estimate the pattern direction, there is no need to transmit the five direction masks and only the directional weighting coefficients for the left view (i.e., ${\eta _{h}^{l}}$, $\eta _{45}^{l}$, ${\eta _{v}^{l}}$, $\eta _{135}^{l}$, and $\eta _{ud}^{l}$) and the right view, need to be estimated at the encoder side and transmitted to the decoder side. Obviously, the overhead bit cost of transmitting the weighting coefficients is negligible in comparison to the bit cost of texture and depth map. In the experimental results section, the term “DDFU” will be used to refer to this proposed full version scheme. In addition, DDFU can be simplified to only transmit the weighting coefficients of the first frame, which will be used for the fusion of all the remaining frames as well. This simplification is possible as the content of each frame does not change significantly, especially for the sequences with slow motion. Based on this observation, the simplified approach can further reduce the amount of transmitted side information with little quality degradation. In the experimental section, the term “DDFU (first frame *η*)” will be used to refer to this simplified scheme.

**Fig. 8:**
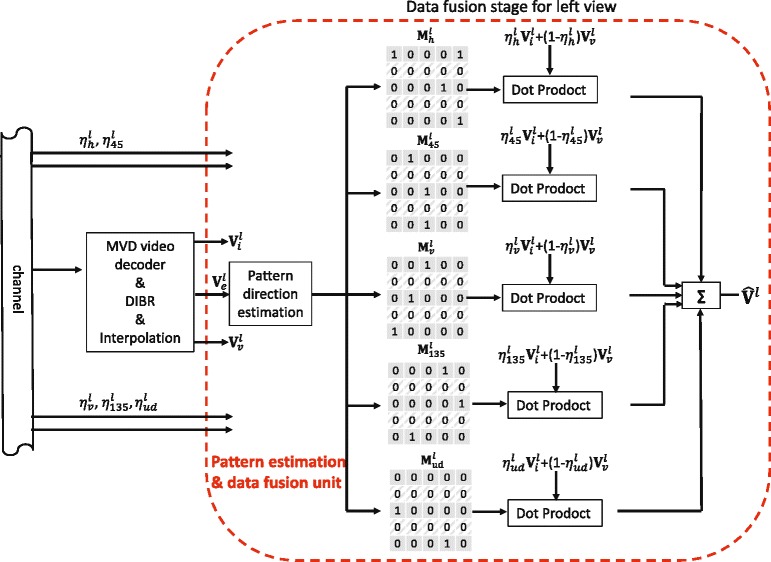
Directional data fusion process. The process of data fusion by directional weighting coefficients and corresponding directional binary masks

Although directional data fusion process happens at both encoder and decoder sides, it has different targets. At the encoder side, it involves the original FR frames to estimate the optimal weighting coefficients for all the directions. Then, as the outputs, these coefficients are sent to the decoder side and used in the fusion process to reconstruct the FR frames. A graphic representation of the proposed directional data fusion process at the decoder side is shown in Fig. 8. By receiving the weighting coefficients and using the same pattern direction estimation process, virtual view pixels and directional interpolated pixels can be fused to generate the recovered frame using Eq. (5).

## Generalization of proposed down/upsampling method

**Fig. 9:**
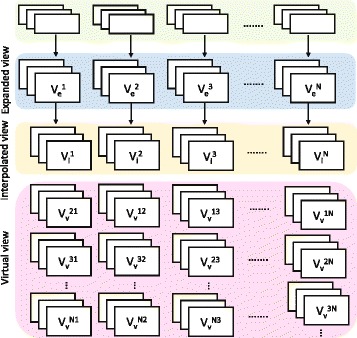
The proposed algorithm for multiview video

The proposed down/upsampling with virtual view-assisted data fusion algorithm can also be applied to MVD video in addition to stereo video. Since in this kind of video more neighboring views and the corresponding depth maps are available, at a given viewpoint, more virtual view versions can be utilized. With the aid of these virtual views, the quality of the final recovered FR views can be considerably improved. As depicted in Fig. 9 in a *N* views multiview video, ${V_{e}^{1}}$ is the expanded view at viewpoint 1 and similarly for ${V_{e}^{2}}$. ${V_{i}^{1}}$ is the directional interpolated view at viewpoint 1, and $V_{v}^{21}$, $V_{v}^{31}$, and $V_{v}^{N1}$ are the virtual views generated from the adjacent views 2,3,*N* at viewpoint 1. In this case, the discarded pixels are recovered by fusing interpolated pixels with one of the available virtual views that gives the minimum differences when compared with original FR pixels. Subsequently, the fusion coefficients are transmitted. In this way, the proposed algorithm can also effectively recover the FR frames.

## Experimental results

To objectively evaluate the performance of the proposed method, several experiments were conducted on public 3D video datasets [25, 26] “Doorflower,” “Kendo,” “Dog,” “Balloons,” “Newspaper,” and “Undo-Dancer”. Some parameters and content characteristics of the testing sequences are listed in Table 1 for reference. For each sequence, both the left and right views had been interlacing and complementary row downsampled with a factor 2 before encoding. JMVC 5.0 [27] was used for compression, and six different QPs, namely 34, 37, 40, 43, 46, and 49, were used to code the texture and depth map sequences. The temporal GOP size and the total number of encoded frames was 8 and 80, respectively, while the delta QP and the differential QP between the base layer and sublayer in hierarchical-B picture structure was set to zero in all layers. The virtual views at the decoder side were rendered using a 1D DIBR technique from one reference view to another view without any postprocessing (i.e., no hole filling).

**Table 1:** The parameters and characteristics of each used sequence

	Size	Camera	Left	Right	Content’s motion
Doorflower	1024×768	Fixed	View10	View08	Moderate
Kendo	1024×768	Moving	View03	View05	Complex
Dog	1280×960	Fixed	View38	View39	Medium
Balloons	1024×768	Moving	View03	View05	Complex
Newspaper	1024×768	Fixed	View04	View06	Simple
Undo-Dancer	1920×1088	Moving	View02	View05	Complex

### Performance evaluation on stereoscopic video

**Fig. 10:**
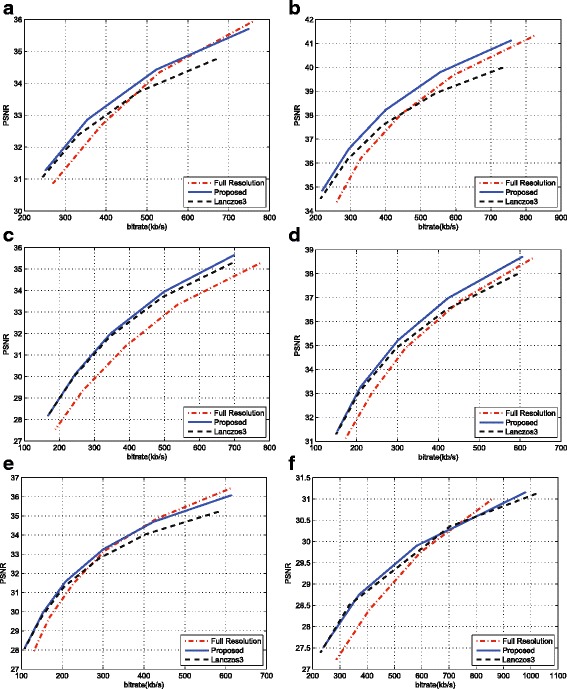
The rate distortion curves. The rate distortion curves for the testing sequences. **a** Doorflower. **b** Kendo. **c** Dog. **d** Balloons. **e** Newspaper. **f** Dancer

The first set of simulations aims to evaluate the effectiveness of the proposed approach by comparing the rate distortion performance with FR video coding approach, filter-based approach, and the state-of-the-art approaches [19, 28]. Figure 10 shows the coding performance of these methods on all of the testing sequences. In the comparison, a 6-tap Lanczos filter has been used at encoder and decoder sides for down/upsampling, respectively, for the filter-based approach. From Fig. 10, the effectiveness of the proposed approach over the matched-filter approach and FR coding at low bit rate can be appreciated for all the testing sequences. The proposed method has higher PSNR results than the matched-filter method, and the maximum PSNR gains can be 0.81 and 0.76 dB on sequences “Undo-Dancer” and “Doorflower”, respectively. This is due to the proposed fusion mechanism that can well preserve the edges. In addition, the better quality of the depth maps is the more contributions the virtual view pixels can make. Generally, the matched-filter approach has good coding performance on the smooth areas; therefore, for the sequences (e.g. “Dog”) containing more smooth areas, it is comparable to the proposed method. However, if more texture is contained in the sequences, the proposed method can be more advantageous over the matched filter method. In summary, the average PSNR gains across different bit rates for all the sequences range from 0.18 to 0.45 dB.

**Fig. 11:**
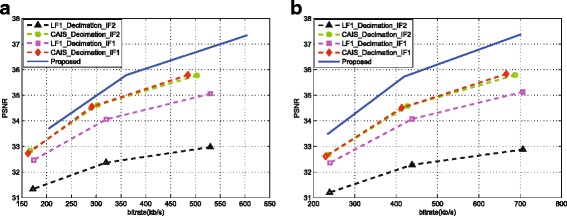
Comparison with [19]. The rate distortion curves for the testing sequences. **a** Doorflower. **b** Laptop, for the proposed approach and [19]

**Fig. 12:**
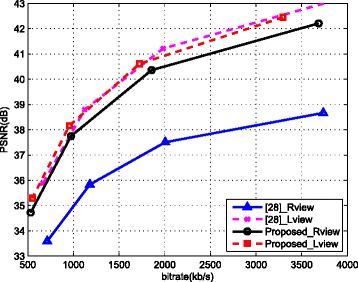
Comparison with [28]. The rate distortion curves for the testing sequence balloons for the proposed approach and [28]

To further evaluate the effectiveness of the proposed method, two fair comparisons with the frame-compatible coding methods in [19, 28]^3^ are carried out by adopting the same testing sequences with the same resolution used in their work, the same coding standard, and the same coding parameters as [19, 28]. The comparison results of [19] are shown in Fig. 11, where LF1 represents the direct downsampling (i.e., even rows in both left and right views are discarded without low pass filtering), CAIS represents the proposed method in [19], and IF1 and IF2 are two interpolation filters with coefficients {1,−5,20,20,−5,1}/32 and {−3,28,8,−1}/32, respectively, as proposed in [19]. The depth sequences used in the proposed DDFU are generated by the method presented in [29], and depth map bit rates have been included in the total bit rate. Indicated by these results, the gain of the proposed method is larger than that of [19]. Figure 12 shows the comparison results of the proposed approach with respect to [28]. In [28], two views are asymmetrically downsampled in frame-compatible coding and the left view has higher quality. Separately comparing the left view and right view, it is obvious that even the left view in the proposed work contains less information (downsampled by factor of 2) than the left view in [28] (downsampled by factor of 8/5); the left view recovery performance of the proposed work is comparable to [28]. Meanwhile, the recovery performance of the right view of the proposed approach is much higher than that in [28]. From the comparison with frame-compatible coding methods, the superiority of the proposed method is due to jointly take into account the features of down/upsampling, the inter-view redundancy, and virtual views.

**Fig. 13:**
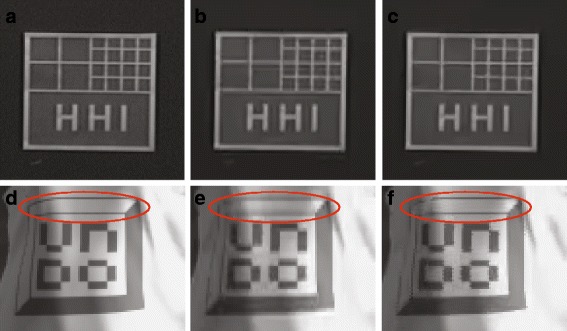
The visual results. Comparison between proposed DDFU method and benchmark method. **a**, **b**, and **c** are the results of Original, Benchmark, and DDFU, respectively, on zoomed-in part of the sequence Doorflower; **d**, **e**, and **f** are the results of Original, Benchmark, and DDFU, respectively, on zoomed-in part of the sequence Undo-Dancer

The visual results of zoomed-in parts of sequences “Doorflower” and “Undo-dancer” are shown in Fig. 13. It is possible to note that the edges recovered by DDFU are sharper than those recovered by the benchmark method. Although the proposed DDFU recovered frame also has some blurred areas, nevertheless, it still achieves a better visual quality than matched-filter-interpolated frame. Figure 13d shows a portion of the original left view of “Undo-Dancer’, and its recovered versions using the matched-filter-based approach and the proposed approach in Fig. 13e, f, respectively. Since the one-pixel-wide edge is difficult to recover properly by using only the surrounding pixels, the advantage of the DDFU method is more obvious in the highlighted areas by a red ellipse in Fig. 13e, f. From this comparison, it can be seen that the proposed approach can recover the one-pixel-wide edge without blurring.

### Performance of each stage of the proposed method

**Fig. 14:**
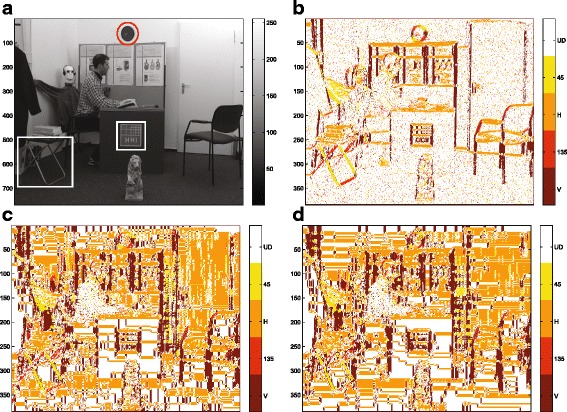
The pattern direction estimation results. **a** Original texture; the pattern direction estimation results on (**b**) original uncompressed texture, **c** compressed texture with QP = 34, and (**d**) compressed texture with QP = 40; the colors *dark red*, *red*, *orange*, *yellow*, and *white* represent vertical, 135° diagonal, horizontal, 45° diagonal, and undefined direction pixels, respectively. (For clearness, the directional estimation results on the discarded pixels are scaled up to the same size as the original texture; their real size is shown on the *y*-axis of each figure)

In this subsection, several experiments have been conducted to validate the necessity and effectiveness of direction estimation and data fusion step in the proposed algorithm. Since the category of the to-be-filled pixels is determined by the estimated texture pattern, accurate pattern direction estimation plays an important role in the fusion process. Therefore, to verify its effectiveness, Fig. 14b shows the pattern estimation result on the uncompressed frame, whereas, Fig. 14c, d shows the estimation results on the compressed frame with *QP*=34 and *QP*=40, respectively. For reference, Fig. 14a shows the original uncompressed texture frame from the “Doorflower” sequence with three highlighted parts containing clear patterns. Different colors are used to distinguish the five directions, so the colors dark red, red, orange, yellow, and white are used to represent vertical, 135° diagonal, horizontal, and 45° diagonal edges and the undefined pattern areas, respectively. In this paper, areas are regarded as undefined pattern areas when *S*_11_/*S*_22_≤*Th* where *Th*=4. The accuracy of the adopted pattern detection algorithm could be appreciated from Fig. 14b, c. By comparing these two figures, the direction estimation results of the three highlighted parts are almost the same. It demonstrates that the accuracy of the pattern estimation is barely affected by the compression distortion.

To show the level of contribution of the virtual views in the fusion stage and how the texture pattern direction influences the fusing process, the average fusion coefficients *η*_*h*_, *η*_*v*_, *η*_45_, *η*_135_, and *η*_*ud*_ of both views are reported in Table 2 for the six testing sequences and different QPs. The smaller the value of *η* is, the more important the virtual view pixels are for the recovery of discarded pixels. In the fusion stage, the contribution of virtual view depends on several factors, such as the adopted DIBR technique and depth map quality. It is worth noticing that even with advanced rendering techniques, the generated virtual view may still face a problem in generating high-quality and aligned texture around depth discontinuous areas, where the adopted directional interpolation can compensate well. From this table, it can be seen that virtual view pixels are more important to recover the pixels with horizontal pattern than other directions. On the other hand, the directional interpolated frame is more important to recover the pixels with vertical pattern. For example, the average *η*_*h*_ for the “Undo-Dancer” sequence at *QP*=34 is 0.11 versus *η*_*v*_=0.97. The *η*_45_, *η*_135_, and *η*_*ud*_ values for the two diagonal patterns and undefined pattern lay in between the horizontal and vertical cases, which are 0.63,0.62,0.19 for *QP*=34, respectively. Moreover, it should be noted that for the sequence “Undo-Dancer” which is a computer graphic sequence and consequently has an accurate depth map, the virtual view pixels provide a greater contribution to the final recovered FR frames, in all five directions, with respect to other sequences. As expected, this contribution is remarkably higher for the horizontal pattern.

**Table 2:** The two-views-all-frames average values of *η*_*h*_, *η*_*v*_, *η*_45_, *η*_135_, and *η*_*ud*_ for each sequence and for different QPs

Doorflower							
QP		34	37	40	43	46	49
Average *η*	*η*_*h*_	0.23	0.24	0.30	0.37	0.47	0.55
	*η*_*v*_	0.97	0.95	0.91	0.89	0.86	0.82
	*η*_45_	0.77	0.76	0.78	0.77	0.75	0.75
	*η*_135_	0.80	0.80	0.79	0.80	0.82	0.79
	*η*_*ud*_	0.58	0.61	0.62	0.65	0.64	0.63
Undo-Dancer							
QP		34	37	40	43	46	49
Average *η*	*η*_*h*_	0.11	0.16	0.23	0.36	0.50	0.58
	*η*_*v*_	0.97	0.98	0.98	0.96	0.95	0.93
	*η*_45_	0.63	0.72	0.79	0.85	0.83	0.80
	*η*_135_	0.62	0.72	0.79	0.82	0.81	0.78
	*η*_*ud*_	0.19	0.28	0.40	0.53	0.62	0.66
Kendo							
QP		34	37	40	43	46	49
Average *η*	*η*_*h*_	0.83	0.81	0.77	0.73	0.68	0.64
	*η*_*v*_	0.95	0.89	0.86	0.81	0.78	0.74
	*η*_45_	0.94	0.91	0.89	0.84	0.77	0.71
	*η*_135_	0.93	0.90	0.87	0.83	0.80	0.75
	*η*_*ud*_	0.86	0.83	0.79	0.75	0.69	0.66
>Balloons							
QP		34	37	40	43	46	49
Average *η*	*η*_*h*_	0.86	0.85	0.82	0.77	0.72	0.65
	*η*_*v*_	0.95	0.95	0.95	0.94	0.91	0.87
	*η*_45_	0.95	0.95	0.93	0.90	0.87	0.82
	*η*_135_	0.95	0.95	0.94	0.91	0.89	0.87
	*η*_*ud*_	0.93	0.93	0.91	0.88	0.84	0.78
Dog							
QP		34	37	40	43	46	49
Average *η*	*η*_*h*_	0.95	0.95	0.92	0.89	0.85	0.77
	*η*_*v*_	1.00	0.98	0.95	0.94	0.89	0.80
	*η*_45_	0.99	0.96	0.94	0.90	0.84	0.78
	*η*_135_	0.99	0.97	0.95	0.93	0.86	0.78
	*η*_*ud*_	0.99	0.97	0.95	0.91	0.90	0.82
Newspaper							
QP		34	37	40	43	46	49
Average *η*	*η*_*h*_	0.89	0.89	0.86	0.85	0.81	0.77
	*η*_*v*_	1.00	1.00	1.00	1.00	0.99	0.96
	*η*_45_	1.00	1.00	1.00	0.98	0.94	0.93
	*η*_135_	1.00	1.00	1.00	0.98	0.96	0.94
	*η*_*ud*_	0.98	0.99	0.98	0.93	0.92	0.89

### Performance evaluation on multiview video

When testing on multiview video, the View1, View3, and View5 of sequences “Kendo” and “Balloons” and the View2, View4, and View6 of sequence “Newspaper” are adopted. For multiview testing, the same codec setting up is used as the two-view testing and after proposed downsampling method, each LR view is in half size of its corresponding FR view. The performance comparison at the decoder side is shown in Table 3. From the reported results, it is possible to note that the proposed down/upsampling method can also work properly in multiview video system. In this case, the highest PSNR gain can be up to 1.19dB for the “Kendo” sequence. Compared with the two-view video case, the PSNR gains of multiview video become higher and the average gain for all the sequences and all QPs is 0.4 db. These gains are obtained due to the availability of multiple virtual view candidates. This ensures that the more suitable virtual view pixels are merged with the interpolated view. Moreover, compared with the average *η* value for two-view testing, in multiview testing, the virtual view has more impacts at low bit rate (Table 4).

**Table 3:** The upsampling performance comparison on multiview video

Kendo							
QP		34	37	40	43	46	49
Bit rate (kb/s)		1073	808	608	476	361	283
PSNR	Lanc	37.12	35.88	34.38	32.79	30.94	29.10
	Pro	38.31	36.71	34.95	33.19	31.26	29.39
*Δ*PSNR		1.19	0.83	0.57	0.40	0.32	0.29
Balloons							
QP		34	37	40	43	46	49
Bit rate(kb/s)		1150	824	577	426	309	245
PSNR	Lanc	36.54	34.97	33.12	31.37	29.54	27.86
	Pro	37.07	35.32	33.36	31.54	29.67	28.00
*Δ*PSNR		0.53	0.36	0.23	0.17	0.14	0.14
Newspaper							
QP		34	37	40	43	46	49
Bit rate(kb/s)		1134	809	572	426	317	253
PSNR	Lanc	34.12	32.90	31.44	29.94	28.23	26.55
	Pro	34.85	33.41	31.77	30.16	28.39	26.67
*Δ* PSNR		0.73	0.51	0.33	0.22	0.15	0.12

**Table 4:** The two-view all-frame average values of *η*_*h*_, *η*_*v*_, *η*_45_, *η*_135_, and *η*_*ud*_ for three multiview sequences and different QPs

Kendo							
QP		34	37	40	43	46	49
Average *η*	*η*_*h*_	0.91	0.89	0.85	0.79	0.71	0.62
	*η*_*v*_	0.99	0.96	0.91	0.87	0.82	0.75
	*η*_45_	0.98	0.94	0.91	0.86	0.81	0.71
	*η*_135_	0.96	0.91	0.89	0.84	0.77	0.72
	*η*_*ud*_	0.95	0.91	0.85	0.77	0.70	0.62
Balloons							
QP		34	37	40	43	46	49
Average *η*	*η*_*h*_	0.94	0.91	0.86	0.82	0.74	0.63
	*η*_*v*_	1.00	0.99	0.95	0.91	0.87	0.80
	*η*_45_	0.97	0.96	0.94	0.89	0.84	0.78
	*η*_135_	1.00	0.98	0.93	0.90	0.84	0.78
	*η*_*ud*_	0.99	0.95	0.92	0.88	0.82	0.74
Newspaper							
QP		34	37	40	43	46	49
Average *η*	*η*_*h*_	0.87	0.86	0.84	0.83	0.78	0.73
	*η*_*v*_	1.00	1.00	1.00	1.00	0.99	0.95
	*η*_45_	1.00	1.00	1.00	0.99	0.96	0.92
	*η*_135_	1.00	1.00	1.00	0.99	0.95	0.92
	*η*_*ud*_	0.99	0.99	0.98	0.96	0.93	0.87

### Performance of simplified method

**Fig. 15:**
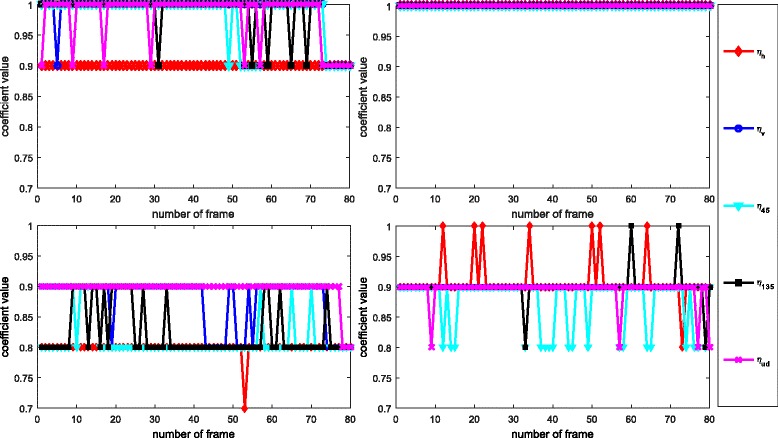
The variance of five *η* coefficients. The pane shows the five coefficients for the sequence “Dog”. The *top left* and *right* figures of the pane are the weighting coefficients of the left and right views, respectively, when QP = 34; the *bottom left* and *right* figures of the pane are the weighting coefficients of the left and right views, respectively, when QP = s46

**Fig. 16:**
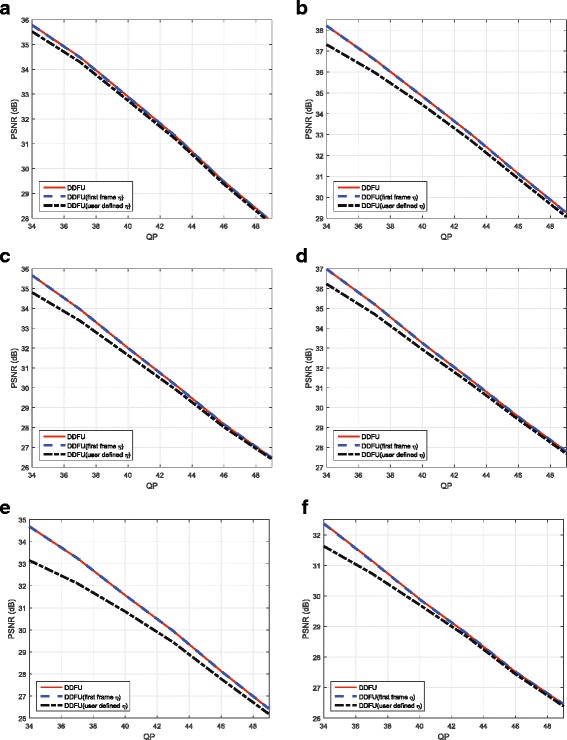
The comparisons of proposed simplified approach. The comparisons of proposed simplified approach with full version approach and user-defined coefficient approach on the testing sequences (**a**) Doorflower, **b** Kendo, **c** Dog, **d** Balloons, **e** Newspaper, and **f** Dancer

In the basic implementation of the DDFU algorithm, the encoder needs to transmit the five weighting coefficients, *η*, for each frame and each view. Clearly, it needs to evaluate them by minimizing (4). However, given that in most cases, there are no major changes in the scene content, it is reasonable to assume that those coefficients do not change very much from frame to frame; hence it is not necessary to evaluate them for each frame. This assumption could be verified by Fig. 15 which shows the trend of the weighting coefficients versus frame number. Thus, one way to reduce the overhead transmission of the proposed approach is using directional weighting coefficients of the first frame for the whole sequence (called DDFU (first frame *η*)). In this approach, the weighting coefficients are only estimated for the first frame and then used for the whole sequence. To verify the effectiveness of this simplified approach, its performance has been compared with the proposed DDFU approach and another simplified approach called in the following DDFU (user defined *η*), the latter adopts user defined coefficients at the decoder side for the whole sequence. The pre-set values for the DDFU (user defined *η*) used are *η*_*v*_=1, *η*_*h*_=0, *η*_45_=0.5, *η*_135_=0.5, and *η*_*ud*_=1 for the left and right views which means that all vertical edges and undefined pattern areas are recovered by the directional interpolation algorithm. All recovered horizontal edges are obtained from the virtual view pixels, and the two diagonal direction pixels are obtained by equally fusing the directional interpolated pixels with the virtual view pixels. The results of this comparison are listed in Fig. 16.

From Fig. 16, it can be seen that DDFU and DDFU (first frame *η*) have almost the same performance for all sequences, which demonstrates the validity and effectiveness of the simplified approach. By comparing the results of DDFU and DDFU (user defined *η*), the importance of adapting the coefficients to the scene content can be appreciated. The results in Fig. 16 show that the performance of DDFU (first frame *η*) are better than that of DDFU (user defined *η*). This is due to the *η* values for the DDFU (first frame *η*) are based on the content of the testing sequence, if the content of the sequence does not vary hugely frame by frame, neither does the value of *η*, while the values of the predetermined *η* are user defined values, which means they do not take the content of the sequence into account. The performance of DDFU (user defined *η*) highly depends on how close the predetermined values are to the frame-by-frame evaluated coefficients.

## Conclusions

In this paper, an interlacing-and-complementary-row-downsampling method is employed on the two adjacent views of a multiview video at the encoder side to reduce the transmitted data. This downsampling method allows the proposed directional data fusion upsampling (DDFU) algorithm to recover the discarded pixels by exploiting the information of the downsampled views and the corresponding virtual views. In the proposed upsampling approach, edge directions around the discarded pixels are estimated by principal components analysis. This information is subsequently used to steer the fusion of the virtual view with the directional interpolated pixels. The aim behind this is to exploit the inter-view redundancy to minimize the overall system distortion, which is a combination of the compression distortion and the distortion introduced by the downsampling process. Therefore, different from filter-based interpolation algorithms, the advantages of virtual views have been exploited by the proposed method. Moreover, it has been shown that the proposed algorithm achieves superior performance in comparison with filter-based interpolation algorithms and the state-of-the-art algorithms. The future work will be devoted to exploiting the temporal correlation in video sequences to control the fusion process.

## Endnotes

^1^ The pixels on the boarder of the frame will be filled by filter-based interpolation without estimating their pattern directions.

^2^ Although using *p*_2_ and *p*_8_ may seem beneficial, the lack of *p*_4_ and *p*_6_ will negatively affect the direction estimation due to the non-symmetric set of pixels. Nevertheless, *p*_2_ and *p*_8_ will be used in pattern estimation of the following discarded pixel, *p*_6_.

^3^ All the results of [19, 28] have been obtained from the author and the paper, respectively.
